# Lysol

**Published:** 1890-12-13

**Authors:** 


### PHARMACY.

### LYSOL.

This is the name given to a new extract of tar prepared by Schülke and Mayr, of Hamburg, a more powerful germicide than carbolic acid or creolin, and possessing the further advantages of constant composition, and of dissolving in alcohol or water in any proportion as a perfectly clear and uniform solution. It appears as a clear brownish yellow oily transparent fluid, with an alkaline reaction, freely mixable with water, to which it does not impart the slightest turbidity. On prolonged exposure to the light and air it assumes a darker hue, though remaining as translucent as before. A 2 per cent. solution in water when used for washing the hands feels like a solution of soap, with an aromatic odour of tar.

